# Amorphous Silica Particles Relevant in Food Industry Influence Cellular Growth and Associated Signaling Pathways in Human Gastric Carcinoma Cells

**DOI:** 10.3390/nano7010018

**Published:** 2017-01-13

**Authors:** Anja Wittig, Helge Gehrke, Giorgia Del Favero, Eva-Maria Fritz, Marco Al-Rawi, Silvia Diabaté, Carsten Weiss, Haider Sami, Manfred Ogris, Doris Marko

**Affiliations:** 1 Department of Food Chemistry and Toxicology, Faculty of Chemistry, University of Vienna, Waehringer Strasse 38, 1090 Vienna, Austria; anja.wittig@univie.ac.at (A.W.); helge.gehrke@gmx.de (H.G.); giorgia.del.favero@univie.ac.at (G.D.F.); efritz@gmx.at (E.-M.F.); 2 Institute of Toxicology and Genetics (ITG), Karlsruhe Institute of Technology (KIT), Campus Nord, Hermann-von-Helmholtz-Platz 1, 76344 Eggenstein-Leopoldshafen, Germany; marco.al-rawi@web.de (M.A.-R.); silvia.diabate@kit.edu (S.D.); carsten.weiss@kit.edu (C.W.); 3 MMCT Laboratory of Macromolecular Cancer Therapeutics, Department for Pharmaceutical Chemistry, Faculty Center for Pharmacy, University of Vienna, Althanstraße 14, 1090 Vienna, Austria; haider.sami@univie.ac.at (H.S.); m.ogris@univie.ac.at (M.O.)

**Keywords:** silica particles, cytotoxicity, proliferation, Ki-67, EGFR and MAPK signaling, gastrointestinal tract

## Abstract

Nanostructured silica particles are commonly used in biomedical and biotechnical fields, as well as, in cosmetics and food industry. Thus, their environmental and health impacts are of great interest and effects after oral uptake are only rarely investigated. In the present study, the toxicological effects of commercially available nano-scaled silica with a nominal primary diameter of 12 nm were investigated on the human gastric carcinoma cell line GXF251L. Besides the analysis of cytotoxic and proliferative effects and the comparison with effects of particles with a nominal primary diameter of 200 nm, emphasis was also given to their influence on the cellular epidermal growth factor receptor (EGFR) and mitogen-activated protein kinases (MAPK) signaling pathways—both of them deeply involved in the regulation of cellular processes like cell cycle progression, differentiation or proliferation. The investigated silica nanoparticles (NPs) were found to stimulate cell proliferation as measured by microscopy and the sulforhodamine B assay. In accordance, the nuclear level of the proliferation marker Ki-67 was enhanced in a concentration-dependent manner. At high particle concentrations also necrosis was induced. Finally, silica NPs affected the EGFR and MAPK pathways at various levels dependent on concentration and time. However, classical activation of the EGFR, to be reflected by enhanced levels of phosphorylation, could be excluded as major trigger of the proliferative stimulus. After 45 min of incubation the level of phosphorylated EGFR did not increase, whereas enhanced levels of total EGFR protein were observed. These results indicate interference with the complex homeostasis of the EGFR protein, whereby up to 24 h no impact on the transcription level was detected. In addition, downstream on the level of the MAP kinases ERK1/2 short term incubation appeared to affect total protein levels without clear increase in phosphorylation. Depending on the concentration range, enhanced levels of ERK1/2 phosphorylation were only observed after 24 h of incubation. Taken together, the present study demonstrates the potential of the tested silica particles to enhance the growth of gastric carcinoma cells. Although interference with the EGFR/MAPK cascade is observed, additional mechanisms are likely to be involved in the onset of the proliferative stimulus.

## 1. Introduction

Besides nanoparticles composed of titanium oxide, aluminum oxide, carbon and other materials, silica nanoparticles (SiO_2_ NPs) are among the most widely produced [1]. The current and potential applications of engineered amorphous SiO_2_ NPs are not restricted to technical fields [1,2], but include also their use in biotechnology, such as for instance as transporters for DNA and drug delivery [3,4,5,6], cancer therapy [7], enzyme immobilization or as biosensors and biomarkers [8,9,10,11]. Eventually, SiO_2_ NPs find use also in cosmetics, e.g., in toothpaste or skin care products, and also within the food sector. In more detail, they can be found as food additives (E551) like anticaking agents in salt, spices or instant soups, as flavor enhancers or food pigments, as coating material in confectionary products and packaging materials or as health supplements [12,13,14,15,16,17].

Thus, the impact on safety regarding the environment and health due to nanoparticle exposure in general and from SiO_2_ NPs in particular, is of great interest, but the scientific knowledge in this respect is relatively limited. Previous studies of SiO_2_ NPs mainly focused on inhalational exposure [1,2,18,19,20]. Influence on other cell systems, for example, on hepatic [21] and various epithelial-like [18,22] carcinoma cells or primary mouse embryonic cells [23] have been determined to a minor extent. For a detailed review on recent toxicological studies of synthetic amorphous silica, see Fruijtier-Polloth [24]. Likewise, only little is known about potential effects on the gastrointestinal (GI) tract after oral uptake of these particles. Therefore, studies on the oral route of exposure are progressively increasing and more data have recently become available.

Several in vitro studies concerning effects of SiO_2_ NPs on gastrointestinal cells are published. In a recent study, Yang et al. [25] considered four different types of SiO_2_ NPs as safe up to a concentration of 100 µg/mL for both, human gastric epithelial (GES-1) and colorectal adenocarcinoma (Caco-2) cells after 24 h of incubation. However, longer incubation periods or higher concentrations induced DNA damage and reduced cell viability. In contrast, Tarantini et al. [26] detected a reduction of cell viability in Caco-2 cells already at a concentration of 32 µg/mL. Furthermore, in another human colon cancer cell line (HT29) 14 nm SiO_2_ NPs induced cyto- and genotoxic effects at a concentration of 10 µg/mL [27].

In a previous study, we demonstrated that SiO_2_ NPs are taken up by HT29 cells and stimulate cell proliferation when incubated in high fetal bovine serum (FBS)-containing media. In contrast, cytotoxic effects were observed when the cells were incubated under low-serum conditions [28]. Taken this as a starting point, we investigated the effects of SiO_2_ NPs under high-serum conditions in a different carcinoma cell line representative of the stomach (GXF251L) established from a gastric signet ring cell carcinoma [29] and further addressed the mechanism of the SiO_2_ particles with respect to cellular proliferation. Hence, the aim of this study was to determine the effects of SiO_2_ NPs with a nominal primary diameter of 12 nm on GXF251L cells mimicking in vitro an oral uptake to some extent and investigating the cytotoxic and proliferation stimulating potential of the nanoparticles by application of a high-content-high-throughput screening assay, as well as, common toxicity/cell growth assays. For purpose of comparison and to figure out if any effects are owed to the nano-scaled size per se, the effects of bigger SiO_2_ particles with a nominal primary diameter of 200 nm were also analyzed with regard to their influences on cell viability and cell death. To further assess potential proliferative mechanisms, the effects of the SiO_2_ NPs on the nuclear proliferation marker Ki-67 were examined by immunocytochemistry. Previous studies indicate that nanoparticle-induced cell proliferation is associated with interferences with the epidermal growth factor receptor (EGFR)/mitogen-activated protein kinases (MAPK) signaling pathways [30,31]. Both signaling cascades, EGFR and MAPK, are well known to be deeply involved in the regulation of cellular processes like differentiation or proliferation [32,33] and were determined by the means of SDS-PAGE/Western blot, immunocytochemistry and quantitative real-time polymerase chain reaction (PCR).

## 2. Results

### 2.1. Particle Characterization

Particles are known to behave differently in dependency not only of their manufacturing process but also with respect to the conditions on how they come in contact with biological material. Hence, particle characterization with regard to the respective incubation conditions is always mandatory for toxicological evaluation [34,35]. Therefore, SiO_2_ NPs suspended in medium containing various FBS-amounts were characterized with regard to their mean particle diameter, particle size distribution (PSD) and suspension stability. For the purpose of comparison, the stock-suspensions with a particle concentration of 1 mg/mL suspended in 9%-FBS containing RPMI 1640 medium, as well as, in double-distilled water were also examined. The mean particle diameters analyzed by nanoparticle tracking analysis (NTA) and the stability of the suspensions measured by ζ-potential shortly after incubation (0 h) and after 24 h are represented in Table 1. The stock suspensions with a concentration of 1 mg/mL pre-suspended in double-distilled water only exhibited mean particle diameters of (224 ± 17) nm after sample preparation and (220 ± 27) nm after 24 h with modal values of 167 ± 19 nm and 188 ± 8 nm, respectively. Hence, a strong agglomeration and aggregation behavior was already observable even though no FBS or cell culture medium were present. The ζ-potentials of (−33.1 ± 6.6) mV (0 h) and (−25.0 ± 5.0) mV (24 h) revealed a quite strong repulsion among the particles while suspended in water and thus, a well stabilized suspension of the particle agglomerates/aggregates. However, incubation of 1 mg particles/mL in high-serum containing medium resulted in a distinct increase of the particle sizes to (336 ± 7) nm and (301 ± 58) nm after 0 h and 24 h, respectively (it may be noted that no modal values are given for FBS-medium-suspended particles due to the formation of several peaks in contrast to a more definite peak when suspended in water). The increase of the mean diameter also corroborates the ζ-potential values of (−11.5 ± 0.3) mV (0 h) and (−11.3 ± 0.4) mV (24 h) revealing strongly reduced particle repulsion compared to the water-suspension resulting in a rapid particle re-agglomeration/-aggregation. The mean diameters of the SiO_2_ particles suspended in cell culture medium at area concentrations of 31.3 and 93.8 µg/cm^2^ exhibited similar values like the respective 1 mg/mL medium stock suspension independent of the contained FBS amounts. The measured ζ-potentials were also comparable, though it seemed that increasing FBS amounts of 0.9% to 2.7% to 9% lead to minimally increased ζ-potential values.

**Figure 1:**
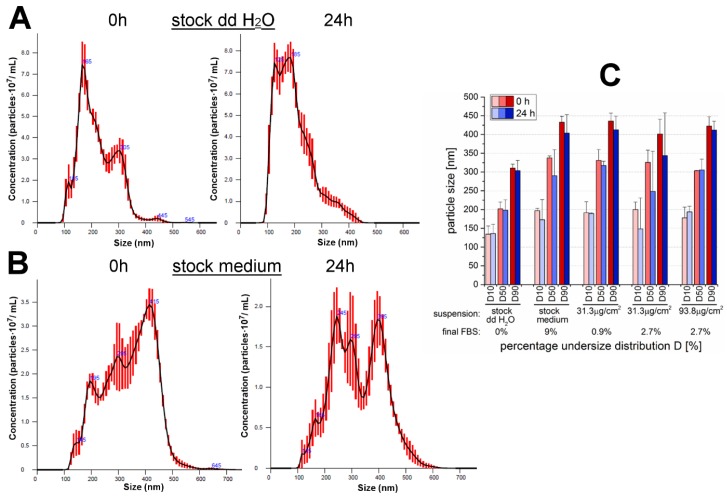
Influence of the suspension medium and the fetal bovine serum (FBS) amount on size distribution of 12 nm SiO_2_ NPs analyzed by nanoparticle tracking analysis after 0 h and 24 h of incubation. Depicted are exemplary particle size distribution profiles (mean ± SEM of five measurements) of 1 mg/mL particle stock suspensions in (**A**) double-distilled water (FBS-free) or (**B**) 9% FBS-containing RPMI 1640 cell culture medium. Please mind the varying ordinate scaling; (**C**) Represented are D10, D50 and D90 values of the particle stock suspensions (1 mg/mL) suspended in either double-distilled water or RPMI 1640 cell culture medium, as well as, the medium incubation suspensions with area concentrations of 31.3 and 93.8 µg/cm^2^. The final FBS amount varied. The D values indicate percentage undersize distribution, for example D10 indicates 10% particles are smaller than the D10 value. This gives indication of the distribution of particle sizes and their corresponding merged particle diameter in [nm] (*n* ≥ 2).

It must be considered that evaluation of the particle size with regard to the mean diameters only is limited. Suspension in double-distilled water resulted predominantly in single peaks whereas particle suspensions in FBS-containing medium clearly exhibited several peaks. For this reason, a detailed look was taken at the percentage particle size distributions. In general, the recorded size distribution curves were in the range of about 100 to 500 nm (suspended in water only) or up to 600 nm (suspended in FBS-containing media), though, for some measurements very small amounts down to diameters of around 75 nm could be found as well. Exemplary PSD curves of both particle stock suspensions are shown in Figure 1A,B. Moreover, software analysis gives D10, D50 and D90 values, which indicate percent undersize, for example D10 indicates 10% particles are smaller than the D10 value. This gives a detailed profiling of the distribution of particle sizes as depicted in Figure 1C. It is clearly visible that 10% of the particles suspended in double-distilled water were smaller than 140 nm whereas the same amount of particles suspended in FBS-containing medium already exhibited sizes around 200 nm. For the FBS-free water-stock suspension the major portion of the particles (D50) was in the range of 200 nm and only 10% were bigger than approximately 310 nm. In contrast to the latter, such strong agglomerates/aggregates with sizes of at least 300 nm were found for up to 50% of the particles suspended in FBS-containing medium (both for stock suspension and incubation suspensions). Here, most of the particles (D90) were not bigger than approximately 400 to 435 nm. Difference between incubation times of 0 h and 24 h were mainly negligible. However, after 24 h particle sizes seemed to be slightly reduced especially for the FBS-stock suspension and its particle dilution with an area concentration of 31.3 µg/cm^2^, but possible significances were concealed by high standard deviations. Altogether, the incubation time or the added amount of either particles or FBS played negligible roles for the particles pre-suspended in FBS-containing medium. Nevertheless, the pre-suspension medium *per se* did influence particle sizes considerably.

### 2.2. Influence of SiO_2_ NPs on Cell Proliferation and Cell Death

In order to investigate the influence of 12 nm SiO_2_ NP on cell viability, together with their influence on necrotic and apoptotic pathways, fluorescence microscopy combined with a computer-based imaging system was deployed. In order to discern nano-specific effects, the 200 nm particles were included for this specific assay as a so-called bulk control. After cell staining and detection, the assay enabled the analysis of viable, early and late apoptotic as well as necrotic cells. Here, three different incubation times (4 h, 24 h, 72 h) were analyzed to differentiate potential short and long term effects.

**Figure 2:**
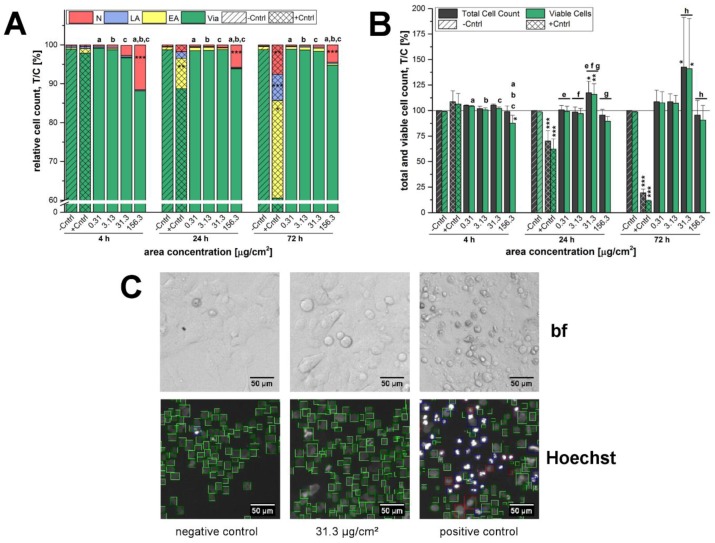
Influence of 12 nm SiO_2_ NPs on cell proliferation and cell death of GXF251L cells monitored microscopically after an incubation period of 4 h, 24 h and 72 h. Staurosporine (800 nM) was applied as a positive control (+Cntrl) resulting in apoptotic and necrotic cell death. (**A**) Viable (Via), early apoptotic (EA) and late apoptotic (LA), as well as, necrotic cells (N) are plotted in relation to the total cell count; (**B**) The depicted bars represent the total cell counts and the respective amount of viable cells. Total cell count was quantified in relation to the negative control (−Cntrl). Cell viability was quantified in relation to the viability of the negative control, as well as, the total cell count of the negative control (T/C [%]). The solid line represents the negative control set to 100%; (**C**) Represented are microscopic bright field (bf) images of the negative and positive control and the incubation sample with 31.3 µg/cm^2^ of 12 nm SiO_2_ NPs after an incubation time of 24 h. Furthermore, the software analysis after detection in the Hoechst-channel is shown distinguishing between viable cells (green), early (blue) and late (violet) apoptotic cells, as well as, necrotic cells (red). Scale bars are equivalent to 50 µm. Statistical analysis was performed by One-way ANOVA followed by Fisher’s LSD test. Significances indicated as * refer to a comparison to the respective negative control (* ≡ *p* ≤ 0.05; ** ≡ *p* ≤ 0.01 and *** ≡ *p* ≤ 0.001; *n* ≥ 3). The same letters indicate a significant difference of these data (in (A): refers to necrotic cells only) with a statistical level of at least *p* ≤ 0.05.

After 4 h of incubation with 12 nm SiO_2_ NPs, only the highest examined concentration of 156.3 µg/cm^2^ exhibited a significant reduction of cell viability of about 12% ± 5% in GXF251L (Figure 2A) due to an enhanced number of necrotic cells. At the same concentration an increase of necrosis was also found after 24 h and 72 h, but did not significantly influence the number of viable cells. Apoptotic events did not seem to play a major role, since only a small number of early and late apoptotic cells were detected during the three incubation times (Figure 2A). In contrast, at a concentration of 31.3 µg/cm^2^ a significant proliferation stimulus was observed (18% ± 10% increase after 24 h and 43% ± 34% after 72 h, respectively) (Figure 2B). Representative bright field images acquired by microscopy and after analysis with the respective software in the Hoechst-channel are shown in Figure 2C.

**Figure 3:**
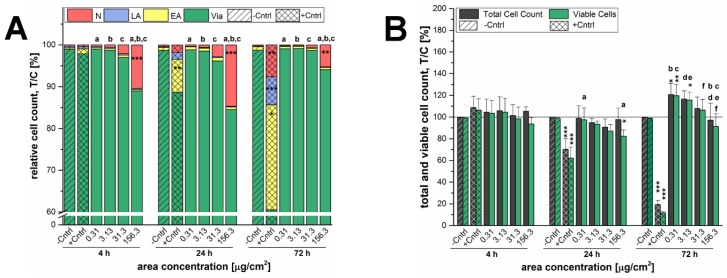
Influence of 200 nm SiO_2_ particles on cell proliferation and cell death of GXF251L cells monitored microscopically after an incubation period of 4 h, 24 h and 72 h. Staurosporine (800 nM) was applied as a positive control (+Cntrl) resulting in apoptotic and necrotic cell death. (**A**) Viable (Via), early apoptotic (EA) and late apoptotic (LA), as well as, necrotic cells (N) are plotted in relation to the total cell count; (**B**) The depicted bars represent the total cell counts and the respective amount of viable cells. Total cell count was quantified in relation to the negative control (−Cntrl). Cell viability was quantified in relation to the viability of the negative control, as well as, the total cell count of the negative control (T/C [%]). The solid line represents the negative control set to 100%. Statistical analysis was performed by One-way ANOVA followed by Fisher’s LSD test. Significances indicated as * refer to a comparison to the respective negative control (* ≡ *p* ≤ 0.05; ** ≡ *p* ≤ 0.01 and *** ≡ *p* ≤ 0.001; *n* ≥ 3). The same letters indicate a significant difference of these data (in (**A**): refers to necrotic cells only) with a statistical level of at least *p* ≤ 0.05.

In Figure 3, the results obtained by the same assay of the SiO_2_ particles with a nominal primary diameter of 200 nm are shown. Here, just like for the 12 nm SiO_2_ NPs, a reduction in cell viability due to necrosis was only observed at an area concentration of 156.3 µg/cm^2^ in GXF251L cells and was at all times significant in comparison to the negative control, as well as, to each other tested concentration (Figure 3A). After 4 h of incubation around 10% ± 5% necrotic cells were found, whereas after 24 h necrosis was most pronounced (15% ± 4%), but was reduced again to 5% ± 4% after 72 h of incubation. Cell death due to early or late apoptosis was negligible as well. The total cell count and cell viability remained nearly constant compared to the negative control after 4 h. The 200 nm particles exerted a significant reduction in cell viability of about 18% ± 6% at the highest examined concentration compared to the negative control and to the lowest examined concentration of 0.31 µg/cm^2^ after an incubation time of 24 h. This effect was mainly due to cell necrosis as described beforehand. After 72 h, a proliferation stimulus was also found for the particles with a bigger nominal diameter. Especially the lower concentrations resulted in an increased total cell count and cell viability. At an area concentration of 0.31 µg/cm^2^ cell growth was significantly increased to 121% ± 11% (total cell count) and 120% ± 10% (cell viability). Incubation with an area concentration of 3.13 µg/cm^2^ stimulated allover cell count of about 17% ± 8% and cell viability was even still significantly increased to 116% ± 7%. The observed proliferation stimulus decreased with increasing particle concentration for both, total and viable cells. At the highest examined concentration, the results were comparable to the negative control and cell viability was significantly decreased in comparison to the other examined particle concentrations (Figure 3B). At this point, another representation of bright field or analysis images is waived.

### 2.3. Influence of SiO_2_ NPs on Mitochondrial Activity, Membrane Integrity, and Cell Growth

**Figure 4:**
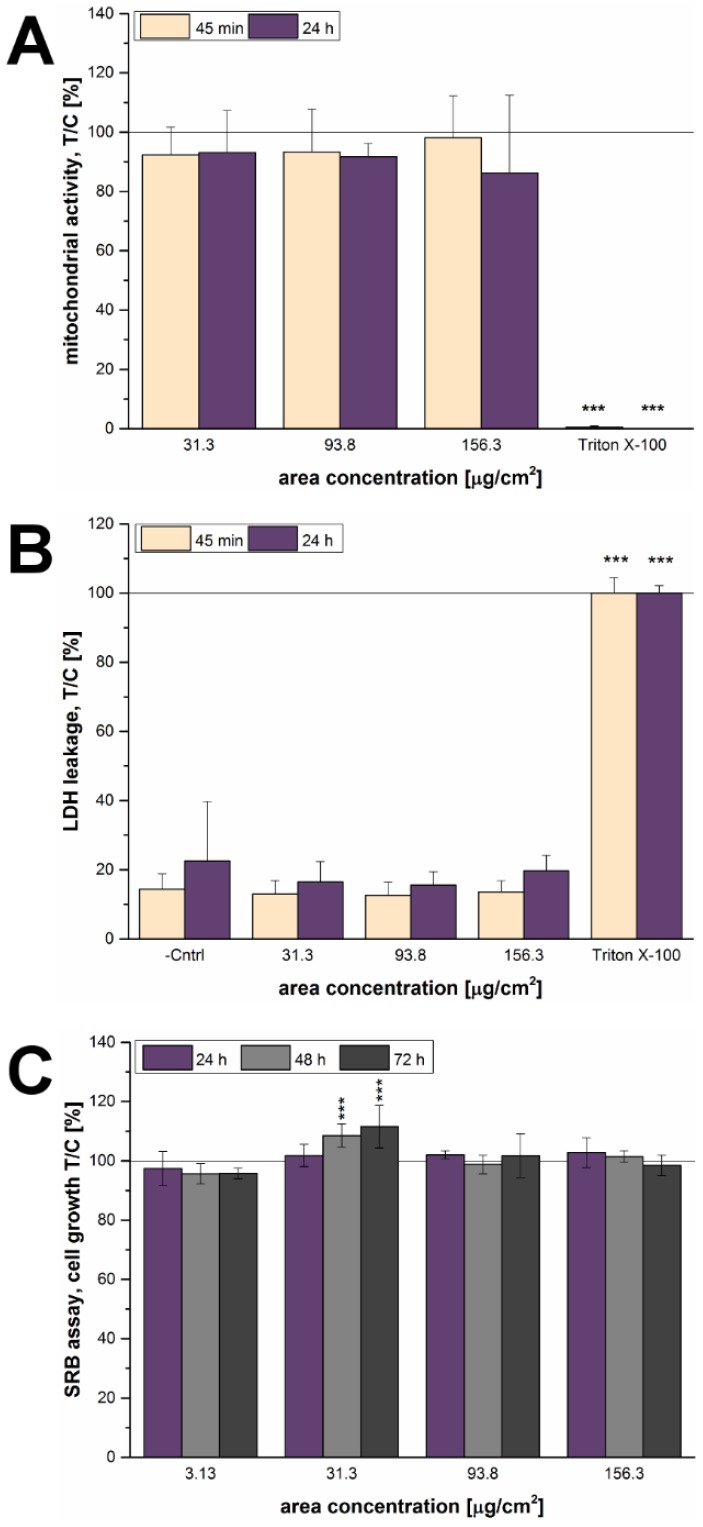
Analysis of the cytotoxic effects of 12 nm SiO_2_ NPs on GXF251L cells. (**A**) Influences on the mitochondrial activity determined by WST-1 assay and (**B**) on the membrane integrity determined by LDH leakage assay after 45 min and 24 h of incubation; (**C**) Growth effects after 24 h, 48 h and 72 h of incubation determined by SRB assay. For WST-1 and SRB assay all effects were quantified in relation to the negative assay control (T/C [%]). For the LDH leakage assay effects were quantified in relation to the positive assay control (T/C [%]). The solid lines represent the respective control set to 100%. Triton X-100 (1% *v*/*v*) was used as positive control. Statistical analysis was performed by One-way ANOVA followed by Fisher’s LSD test. Significances indicated as * refer to a comparison to the respective negative control (* ≡ *p* ≤ 0.05; ** ≡ *p* ≤ 0.01 and *** ≡ *p* ≤ 0.001; *n* ≥ 3).

Since only limited effects on cytotoxicity were observable in the microscopy-based assay, further experiments were performed to investigate the potential for toxicity of 12 nm SiO_2_ NPs on the gastric cells. For this purpose, three classical cytotoxicity assays assessing different read-outs i.e., mitochondrial activity, membrane integrity and protein content were performed, namely WST-1 assay, LDH release assay, and SRB assay, respectively. After 45 min of incubation SiO_2_ NPs did not exert any effect on mitochondrial activity or membrane integrity. Similarly, even after 24 h no appreciable effect was detectable (Figure 4A,B).

In addition, the SRB assay was performed to provide information about the potential of SiO_2_ NPs to stimulate/inhibit cellular growth. Since the protein content serves as an endpoint of measurement, long-term incubations of at least 24 h were necessary and short-term endpoints were not considered relevant for the purpose of the study. Interestingly, in line with previous results shown above a time-dependent and significant increase of growth was observed for the gastric cells at a SiO_2_ NP concentration of 31.3 µg/cm^2^. The effect was visible after 48 h of incubation and persisted up to 72 h (Figure 4C).

### 2.4. Influence of SiO_2_ NPs on the Proliferation Marker Ki-67

**Figure 5:**
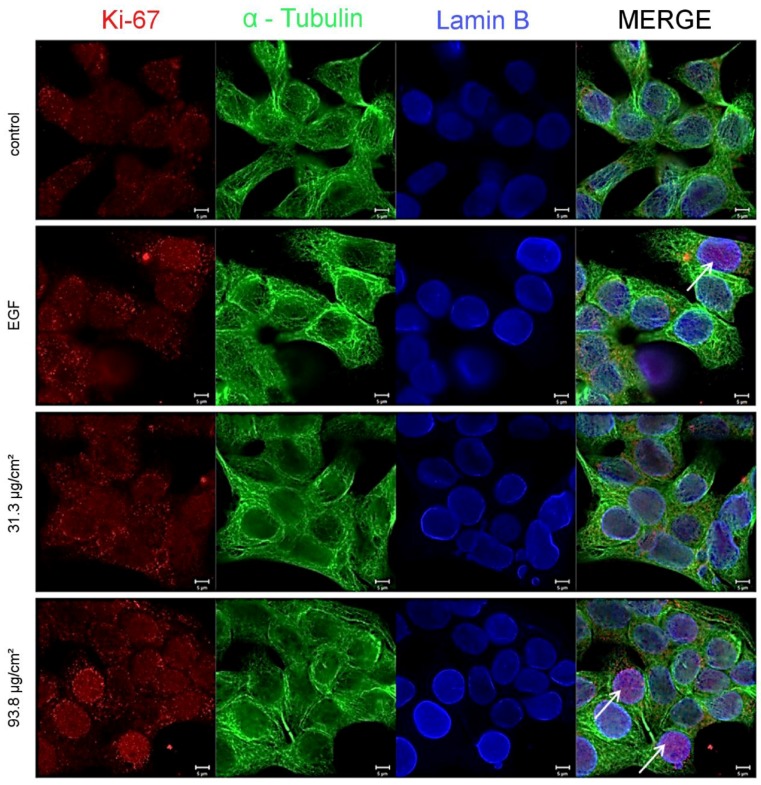
Intracellular levels and localization of Ki-67. Representative images of GXF251L cells treated with 12 nm SiO_2_ NPs for 24 h after immunofluorescence staining of Ki-67 (**red**), α-Tubulin (**green**) and Lamin B (**blue**). White arrows indicate enhanced presence of Ki-67 in the nucleus. Scale bars are equivalent to 5 µm.

Since SiO_2_ NPs proved to have a considerable effect on cell growth, as a further step, their effect on the biomarker of cellular proliferation, Ki-67 was examined. For this reason, immunocytochemical experiments were performed. After 24 h of incubation with SiO_2_ NPs at the area concentrations of 31.3 and 93.8 µg/cm^2^, a concentration-dependent effect on the nuclear levels of Ki-67 was visible (Figure 5, Ki-67, red). In particular, a significant increase was observed in the nuclear region (identified by the nuclear protein Lamin B, blue, Figure 5) after incubation with the highest concentration and for the positive control EGF (Figure 5, white arrows).

**Figure 6:**
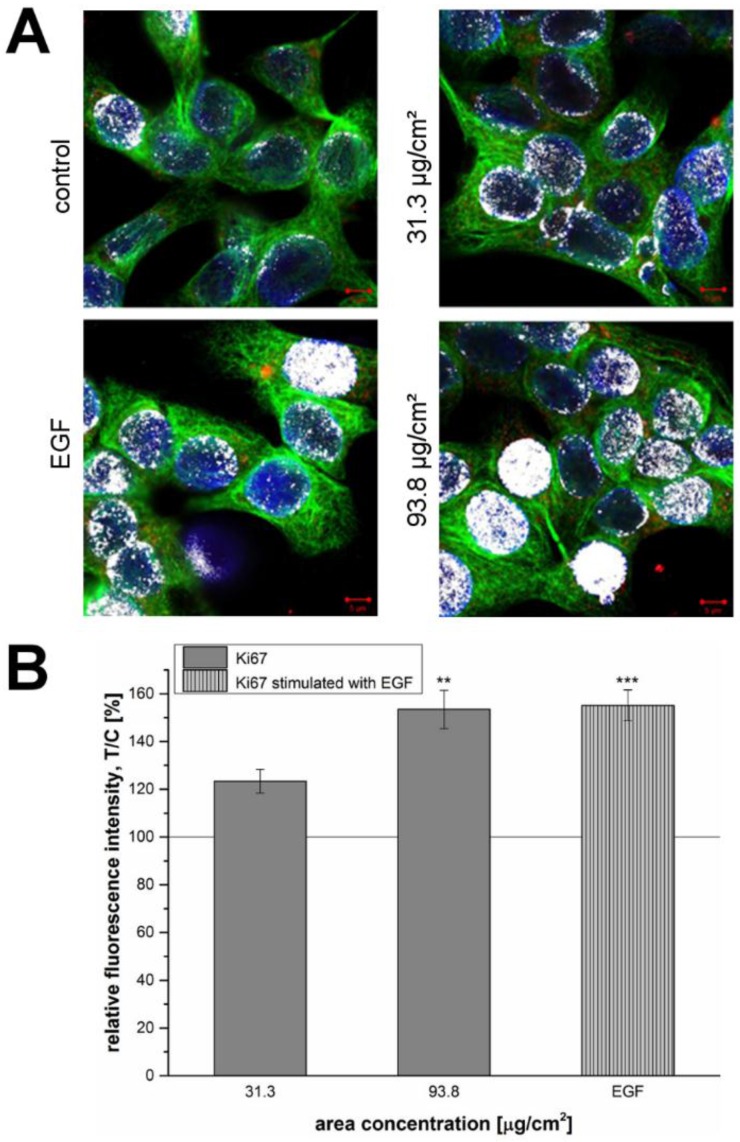
(**A**) Representative appearance of Ki-67 and Lamin B in GXF251L cells treated with 12 nm SiO_2_ NPs for 24 h after the application of the co-localization tool on the images. White fields represent the co-localization of Ki-67 (red) and Lamin B (blue). α-Tubulin is presented in green. Scale bars are equivalent to 5 µm; (**B**) Quantification of the red fluorescence associated with Ki-67 nuclear staining. [%]). The solid line represents the negative control set to 100%. Statistical analysis was performed by Student’s t-test. Significances indicated as * refer to a comparison to the samples treated with SiO_2_ NP concentration of 31.3 µg/cm^2^. (** ≡ *p* ≤ 0.01 and *** ≡ *p* ≤ 0.001; 31.3 µg/cm^2^ *n* = 66 nuclei; 93.8 µg/cm^2^ *n* = 72 nuclei; EGF *n* = 67 nuclei).

In order to specifically detect the signal of Ki-67 in the nucleus co-localization analysis was performed taking nuclear Lamin B as reference (Figure 6A). In this case, the white areas represented the co-localization of Ki-67 and Lamin B and were visualized by color merging (depicted in white). The effect was evident after incubation of GXF251L cells with EGF and SiO_2_ NPs at 93.8 µg/cm^2^. After the performance of three independent experiments, Ki-67 staining was quantified (Figure 6B). Here, a concentration-dependent and significant increase of the proliferation marker was observed for GXF251L cells treated with SiO_2_ NPs concentrations of 93.8 µg/cm^2^.

### 2.5. Influence of SiO_2_ NPs on the Epidermal Growth Factor Receptor Expression

**Figure 7:**
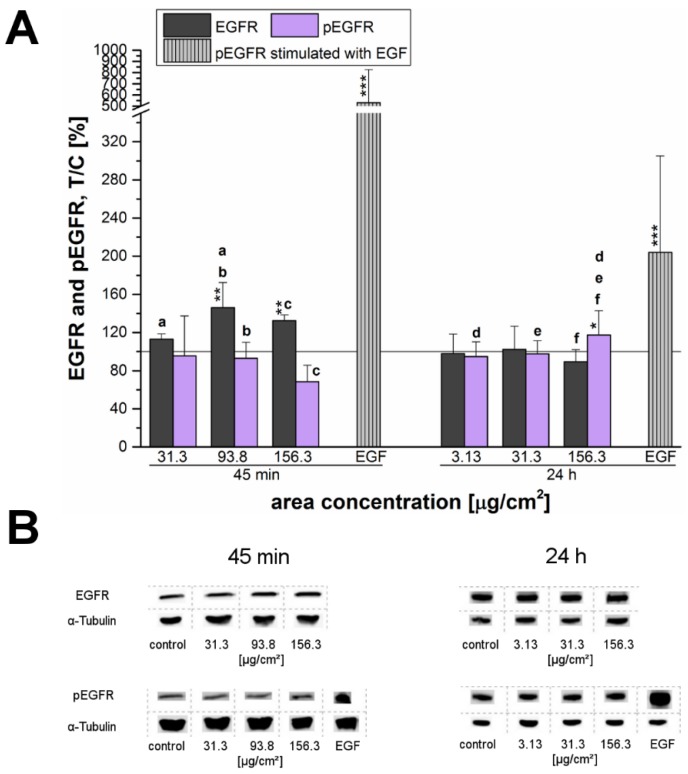
Endogenous epidermal growth factor (EGF) receptor levels and their activation status (pEGFR) in GXF251L cells after 45 min and 24 h of incubation with 12 nm SiO_2_ NPs. EGF was applied as a positive control for phosphorylation of EGFR. (**A**) The relative amount was quantified as arbitrary light units in relation to the negative control (T/C [%]). The solid line represents the negative control set to 100%; (**B**) Representative images of a Western blot experiment. α-Tubulin was used as loading control and detected on the same membrane. Statistical analysis was obtained by one-way ANOVA followed by Fisher’s LSD test. Significances indicated as * refer to a comparison to the respective negative control (* ≡ *p* ≤ 0.05; ** ≡ *p* ≤ 0.01 and *** ≡ *p* ≤ 0.001; *n* ≥ 3). The same letters indicate a significant difference of these data with a statistical level of at least *p* ≤ 0.05.

In order to elucidate the molecular mechanisms that are sustaining the SiO_2_ NP induced cell proliferation Western blot experiments were performed. Previous studies performed on other cell lines [28] suggested the involvement of the EGFR pathway in nanoparticle-induced cellular stimulation, so the expression of the receptor was analyzed as a first step. After an incubation time of 45 min, SiO_2_ NPs were found to significantly enhance the endogenous total EGFR levels in a concentration-dependent manner. This effect started progressively with an increase of 113% ± 6% at a SiO_2_ NP concentration of 31.3 µg/cm^2^. The receptor levels reached their highest value with 146% ± 26% at 93.8 µg/cm^2^ of NPs and then slightly decreased to 132% ± 6% at the highest examined concentration whereas after 24 h of incubation the total receptor levels remained constant (Figure 7A,B). However, in contrast to the increase of the total EGFR level a significantly decreased EGFR phosphorylation was determined at the NP concentrations of 93.8 and 156.3 µg/cm^2^. Long-term incubation revealed a significantly enhanced EGF receptor phosphorylation of 117% ± 25% at the highest concentration (156.3 µg/cm^2^) in comparison to the negative control and to the other examined concentrations, but did not influence the endogenous receptor levels (Figure 7A,B).

### 2.6. Influence of SiO_2_ NP on the Extracellular Signal-Regulated Kinases 1/2

**Figure 8:**
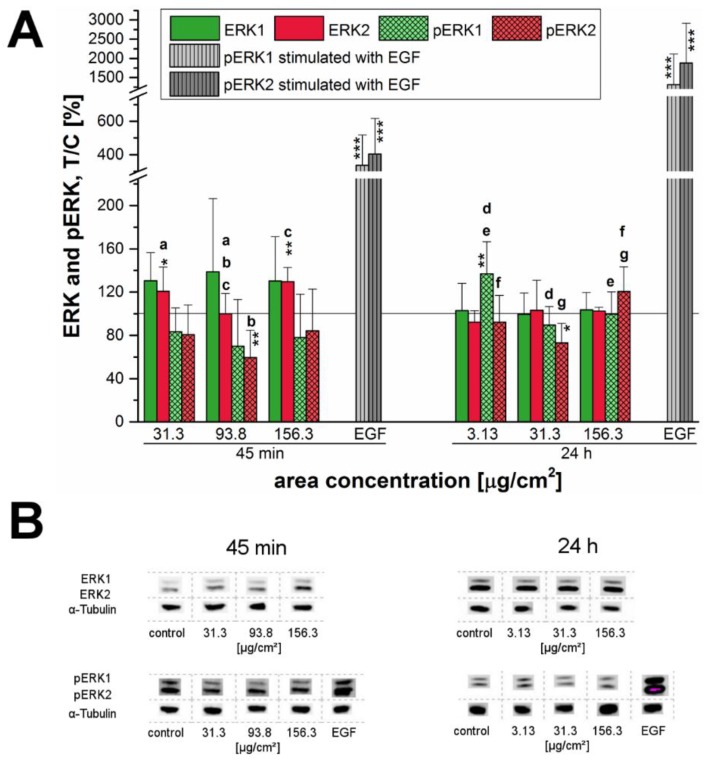
Endogenous ERK1/2 protein levels and their activation status (pERK1/2) in GXF251L cells after 45 min and 24 h of incubation with 12 nm SiO_2_ NPs. EGF was applied as a positive control for phosphorylation of ERK1/2. (**A**) The relative amounts were quantified as arbitrary light units in relation to the negative control (T/C [%]). The solid line represents the negative control set to 100%; (**B**) Representative images of a Western blot experiment. α-Tubulin was used as loading control and detected on the same membrane. Statistical analysis was performed by One-way ANOVA followed by Fisher’s LSD test. Significances indicated as * refer to a comparison to the respective negative control (* ≡ *p* ≤ 0.05; ** ≡ *p* ≤ 0.01 and *** ≡ *p* ≤ 0.001; *n* ≥ 3). The same letters indicate a significant difference of these data with a statistical level of at least *p* ≤ 0.05.

Since the analysis of the EGFR expression revealed an effect of 12 nm SiO_2_ NPs on this signaling pathway, further experiments were performed in order to verify if other components of this cascade could also be affected. To this end the effect on the MAPK—originally called extracellular signal-regulated kinases 1/2 (ERK1/2)—was studied. After short-term incubation, phosphorylation of ERK1/2 seemed to be impaired in general and a significant decrease was especially observed for pERK2 of about 59% ± 25% at a SiO_2_ NP concentration of 93.8 µg/cm^2^ (Figure 8A,B). The endogenous total kinase levels increased strongly of about 20% to 39% except for extracellular signal-regulated kinase 2 (ERK2) at a concentration of 93.8 µg/cm^2^ yielding a significant difference not only to its decreased activation status but also to the ERK2-levels at the tested concentrations of 31.3 µg/cm^2^ and 156.3 µg/cm^2^. In contrast, after 24 h the total ERK1/2 levels remained constant, but phosphorylation varied. Here, activation of ERK1 rose suddenly to a significant value of 137% ± 30% at a concentration of 3.13 µg/cm^2^. Phosphorylation of ERK2 seemed to be slightly but significantly inhibited at a concentration of 31.3 µg/cm^2^ but was activated again by around 21% ± 23% at the highest examined concentration of 156.3 µg/cm^2^ exhibiting a significant difference to both of the other tested concentrations (Figure 8A,B).

### 2.7. Evaluation of Molecular Mechanisms Sustaining the EGFR Increase Triggered by SiO_2_ NPs

**Figure 9:**
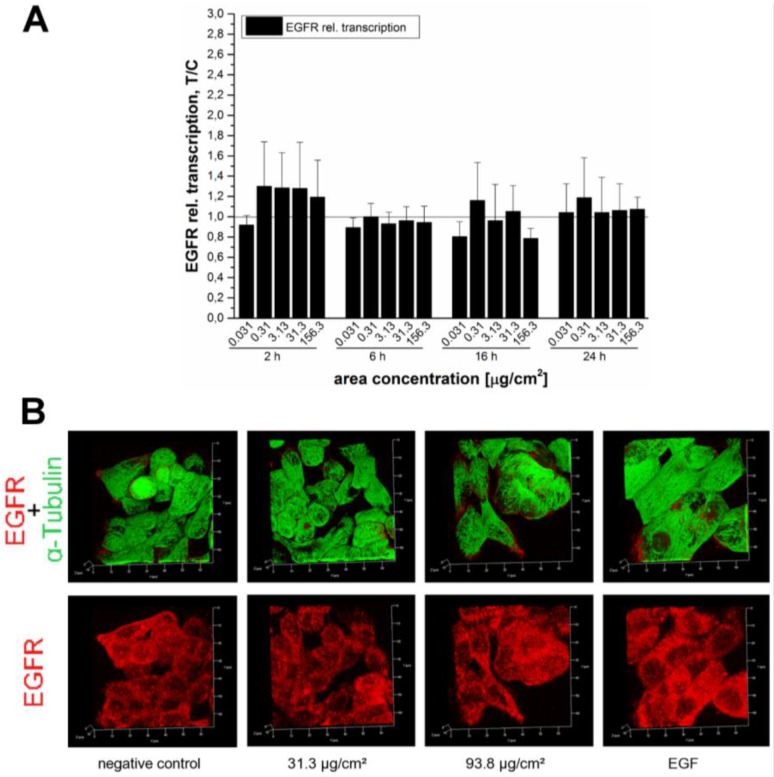
Investigation of the molecular mechanisms influencing the EGF receptor at the cellular level by analysis of its relative mRNA transcription rates and its localization in GXF251L cells. (**A**) Relative gene transcription rates of the EGFR after incubation with 12 nm SiO_2_ NPs for 2 h, 6 h, 16 h and 24 h. The depicted bars represent the relative transcription rates in relation to the negative control (solid line) after normalization to β-actin and GAPDH expression (T/C [%]) (*n* ≥ 3); (**B**) 3D reconstruction of the appearance of the EGFR localization (red) and tubulin cytoskeleton (green) after immunocytochemical analysis. Represented are the results of the negative control, the incubation with 31.3 µg/cm^2^ and with 93.8 µg/cm^2^ of 12 nm SiO_2_ NPs after 45 min of incubation and EGF stimulation as positive control. Scale bar distances are expressed in 10 µm.

Since the growth stimulation pathway seems to be triggered by the activation of the EGFR, further investigations were carried out to study the molecular mechanisms related to this event. As a first step, it was evaluated if the enhanced total EGFR levels were due to increased EGFR transcription rates. For these studies four different incubation times were selected, 2 h, 6 h, 16 h and 24 h, respectively. Interestingly, none of the tested concentrations of the SiO_2_ NPs did increase the EGFR transcription rates (Figure 9A). Since the transcription of EGFR did not seem to be affected by SiO_2_ NPs incubation, the localization/distribution of the receptor in the GXF251L cells was investigated by immunocytochemistry. In line with the Western blot results, after 45 min of incubation in negative controls and in cells incubated with 31.3 µg/cm^2^ SiO_2_ NPs, the levels but also the distribution of the receptor appeared to be relatively homogenous. In contrast, in cells incubated with 93.8 µg/cm^2^ SiO_2_ NPs there seemed to be an increase of the signal related to the presence of the receptor. α-tubulin was co-stained for a better identification of the cellular shape and its appearance remained constant under all applied experimental conditions (Figure 9B).

## 3. Discussion

The objective of this in vitro study was to investigate the effects of amorphous SiO_2_ NPs with a nominal primary diameter of 12 nm on a human gastric cancer cell line. For this purpose, the gastric cell line GXF251L and its responses to SiO_2_ NPs including the influences on viability and possibly related signaling cascades were examined. An extensive description of the physicochemical properties and the cellular uptake of the tested particles has been published previously by Gehrke et al. [28]. Thereby, the influence of, inter alia, the same particles on HT29 cells cultured in DMEM under high and low serum conditions was examined and was considered as starting point of the paper at hand. In general, a particle characterization under the applied incubation conditions is essential for a toxicological assessment [34,35]. For the present study, the examination of a different cell line (GXF251L) was accompanied by the usage of another cell cultivation medium. For this reason, and because of the availability of an innovative method like NTA, the behavior of the particles of interest was characterized with regard to particles size, PSD and suspension stability (Table 1 and Figure 1). In general, the method applied for the determination of particle diameters and PSD is dependent on the particle properties, as well as, the compounds contained in the suspension medium, especially FBS. The examined 12 nm SiO_2_ NPs are known as polydisperse particles exhibiting a strong agglomeration and aggregation behavior and a broad PSD not only when suspended in FBS-containing medium but also presumably already due to their manufacturing process (flame synthesis) and their physico-chemical properties [24,28,36,37]. Hence, the application of methods like dynamic light scattering (DLS), small-angle X-ray scattering etc. was very limited with regard to the given incubation conditions. For example, with DLS the mean particle size is biased towards bigger particle diameters since the light is scattered more intense with increasing particle sizes giving only inappropriate results [38,39]. NTA belongs to the state-of-the-art methods for the determination of particle size and PSD of such complex samples [37,38,39]. Moreover, nanoparticle tracking analysis (NTA) tracks and analyses single particles thereby giving a detailed and highly resolved size profile of the nanoparticle population.

For all the cell-based experimental conditions, 9% FBS was included in the cell culture medium in order to maintain physiological conditions and habitual cell growth. The only exceptions were made for the cell-free particle characterization studies due to limitations of the applied particle size determination method, NTA, with regard to high FBS amounts (for more details see Section 2.1.). Therefore, size analysis was performed with the highest necessary amount of FBS, which was 2.7% for a particle area concentration of 93.8 µg/cm^2^ aiming to prevent high background intensities, as well as, strong particle flares and thus, deviating results. Only the medium stock suspension was accepted for analysis with 9% FBS.

With NTA, the particle suspension in water showed the formation of agglomerates and aggregates and the modal values were comparable to the previously performed DLS measurements of the same 12 nm SiO_2_ NPs by Gehrke et al. [28]. While for the latter mentioned DLS measurements no suitable data could be obtained even in low FBS-containing medium, a more explicit statement was possible by means of NTA applied for the present study. Thereby, particles suspended in medium were strongly agglomerated/aggregated even when incubated in low amounts of FBS. This was also proved by the results of the ζ-potential. The values of the stock suspension prepared in double-distilled water were still within the threshold to be claimed as a stable suspension exhibiting only a slight increase of the agglomeration/aggregation status after 24 h of incubation. In contrast, SiO_2_ NPs suspended in FBS-containing medium were marked by increased particle repulsion compared to the water sample and thus, may have been subject to a rapid re-agglomeration/-aggregation. This behavior was comparable within the analyzed FBS-medium-suspensions and no significant influences due to the particle concentration, the amount of FBS or the incubation time seemed to be observable. Altogether, it was already confirmed by Gehrke et al. [28] that the 12 nm SiO_2_ NPs were sintered beforehand according to the TEM images. Despite the dispersion process by sonification, the particles were strongly agglomerated/aggregated when suspended in FBS-containing cell culture medium. The results are in accordance with former studies of the same particles in cell culture medium containing FBS and it is assumed that the adsorption of serum proteins is responsible for the increase in size [25,28]. Even though studies are controversial with regard to the particle behavior due to the addition of cell culture medium and/or FBS [28], it can most likely be concluded that the allover suspension composition was responsible for the enhanced agglomeration and aggregation of the SiO_2_ particles. Compared with the results of Gehrke et al. [28] for the DMEM-suspended 200 nm particles the secondary diameters of the 12 nm particles suspended in RPMI 1640 medium with ranges around 300 nm due to formed agglomerates/aggregates are even about 100 nm bigger.

Strictly speaking, the 12 nm SiO_2_ NPs cannot be designated as nanoparticles anymore, which is more or less a matter of definition. The common perception is that nanomaterials possess at least one dimension with a maximum range of 100 nm [34,40]. Hence, the term nanoparticle only applies to the primary particles with a nominal diameter of 12 nm, whereas the secondary particles exhibited diameters in the range of approximately 100 nm to 500 or 600 nm.

It must be considered that the size measurements were performed at a physiological pH due to method limitations. However, according to Peters et al. [41] these results seem to resemble the gastric conditions. The working group mimicked the human digestion with the help of an in vitro model and found, depending on the food matrix, that mainly no nano-scaled SiO_2_ at all or only small amounts were present at the gastric digestion stage. The formation of large agglomerates was probably due to the low pH and the high concentrations of electrolytes in the gastric fluid whereas in the previous (saliva) and the subsequent (intestine) digestion stages significantly more nano-sized silica particles were found [41].

For the present study, different incubation times were applied depending on the used assay. Short time effects and especially the relative short residence time of food in the stomach were considered by incubation times of 45 min to 4 h. Furthermore, long term effects, e.g., due to repeated uptake of SiO_2_ NPs were taken into account. Especially for the examination of cell growth or proliferation related effects, incubation times of at least 24 h were necessary since the doubling time of the GXF251L cells of about 32 h needed to be considered as well [42].

A microscopy-based determination of total cell counts including the distinction between viable cells and different types of cell death was deployed as initial assessment of the cytotoxic potential of the 12 nm SiO_2_ NPs. Here, independently of the incubation time the highest applied concentration of SiO_2_ NPs exerted necrotic effects whereas cell death due to apoptosis was negligible in general (Figure 2A). The concentration of 156.3 µg/cm^2^ was in fact primarily selected to obtain an overload concentration of the test system and hence to ensure the detection of cellular effects under extreme stress levels in a range even up to physiologically concentrations less likely to be achieved by exposure to nano-silica via food [25,43,44]. As a consequence, necrosis resulted in a decrease of the viable cell number, which was significant only after short term incubation of 4 h (Figure 2A). This peculiar behavior seems to suggest that the high amount of particles acutely impairs cell viability but at later time points exposed cells are able to handle the administered particles and recover. Comparable results were found for a human keratinocyte cell line exposed to 50 nm silver nanoparticles, both, acutely and chronically [45]. In contrast, GXF251L cells displayed a distinct proliferation stimulation at a concentration of 31.3 µg/cm^2^ after 24 h and 72 h of incubation. Interestingly, similar results were found for the SiO_2_ particles with a bigger nominal primary diameter of 200 nm (Figure 3). Necrotic GXF251L cells were also only found for the highest examined concentration resulting in reduced cell viability and this effect was diminished after 72 h of incubation. As well, cell proliferation was stimulated but not until 72 h of incubation and only for the lowest examined concentration in a decreasing manner with increasing particle concentrations. Consequently, the effects causing cell death, as well as, cell proliferation are present for both SiO_2_ particle preparations independently of the nominal particle diameter.

In order to explore the effects of the 12 nm SiO_2_ NPs in detail, their cytotoxic potential was determined as a next step. The particles did not exert any effects on the mitochondrial activity (WST-1 assay) or membrane integrity (LDH assay) of GXF251L cells, neither after 45 min nor after 24 h of incubation (Figure 4A,B). Interestingly, the SRB assay showed a stimulation of growth at a concentration of 31.3 µg/cm^2^ after 48 h and 72 h of incubation (Figure 4C), which is in accordance with the increase of cell number detected with the microscopy-based assay. Though, a growth stimulus after 24 h was not detectable in the latter assay, which might be due to the different endpoints analyzed by the two assays and the assumption that the microscopy-based assay might be more sensitive than the SRB assay. A similar behavior regarding a growth stimulus was already described for the same particles in HT29 colon cells at a concentration of 31.3 µg/cm^2^ and also at 93.8 µg/cm^2^ [28]. In both cases, differences between the results of the WST-1 and SRB assay suggest that the behavior of NPs cannot be easily categorized and described by one single assay [46,47]. In another study, 20 nm SiO_2_ NPs also exerted slight growth stimulating effects on the gastric cancer cell line MGC80-3 after 24 h detected by MTT assay [48]. However, longer incubation times, as well as, particle concentrations of more than 300 µg/mL lead to significant cytotoxic effects towards MGC80-3 cells.

As a consequence for the present study, a mechanistic approach was carried out with respect to the underlying mechanism of the proliferating effects of the SiO_2_ NPs. Therefore, the cellular proliferation marker, Ki-67 was evaluated by immunocytochemistry. Ki-67 detects a nuclear antigen which is only found in proliferating cells and is absent in resting cells [49]. A concentration-dependent increase of the proliferation marker could also be demonstrated at the SiO_2_ NPs concentrations of 31.3 µg/cm^2^, as well as, 93.8 µg/cm^2^ after 24 h of incubation (Figure 5 and Figure 6).

Furthermore, the phosphorylation of the ERK1/2 and its upstream regulator, the EGFR, were examined after exposure of cells to the 12 nm SiO_2_ NPs (Figure 7 and Figure 8). The EGFR (also known as ErbB1 and HER1 in humans) is a membrane spanning cell surface receptor [50] and plays an important role in cell adhesion, motility, signal transduction, cell division, differentiation and is mediating cellular proliferation, as well as, cell-to-cell communication in mammalian cells [32,51,52]. Previously, it was demonstrated that the induction of nanoparticle-dependent cell proliferation is associated with the activation of the EGFR signaling cascade with a subsequent proliferation-specific ERK1/2 activation, whereas an EGFR-downstream activation of the c-Jun NH_2_-terminal kinases1/2 (JNK1/2) is specific for apoptosis [30]. Furthermore, it was found that the MAPK/ERK1/2 signaling pathway is a key component of pathways controlling cell-cycle processes, differentiation and proliferation, as well as, cell death [28,31,33,53]. Kim et al. [54] as well observed a brief activation of the ERK1/2 pathway followed by an increase in cell proliferation in human adipose tissue-derived stem cells (hADSCs) after incubation with SiO_2_ NPs (diameters in the range of 50–120 nm).

In the present study, a connection between cell proliferation and activation of the EGFR and ERK1/2 signaling pathways was assumed and therefore investigated in more detail. After short-term incubation of 45 min, the endogenous EGFR levels increased especially at high incubation concentrations. This behavior seemed to suggest that EGFR-trafficking was obstructed by the high amount of the applied particles, thus interfering with EGFR homeostasis. However, this hypothesized influence due to a particle overload only seemed to be a matter of short duration—longer incubation of 24 h resulted in a normalization of the endogenous levels comparable to those of the negative control (Figure 7).

After 24 h, the endogenous levels of both, EGFR and ERK1/2 remained constant and only for the EGFR a slight but significant activation was observed at the highest examined concentration (Figure 7A). Interestingly, phosphorylation of ERK1 significantly increased at a low concentration of just 3.13 µg/cm^2^. However, ERK2 did not seem to be affected here—in contrast, its phosphorylation seemed to be declining. Thus, after 24 h of incubation a consistent activation signal for EGFR and downstream for ERK1/2 could only be confirmed for the overload concentration. Obviously, the tested silica particles seem to influence the EGFR/MAPK signaling pathway in a time-dependent manner with regard to the endogenous receptor levels as well as the receptor activation. Comfort et al. [45] associated an observable increased Ki-67 expression, as well as, an activation of the EGFR/MAPK signaling cascade in human keratinocytes to augmented stress and altered functionality of human keratinocytes. However, the results could be assigned to chronic administration of very low silver nanoparticles-concentrations in contrast to an acute exposure of 24 h.

In order to verify whether the endogenous EGF receptor enhancement after 45 min was due to an increased transcription rate or the result of impaired receptor trafficking due to particle overload, the mRNA expression of EGFR was examined. The relative transcriptions rates were measured after several incubation periods of 2 h, 6 h, 16 h and 24 h to capture the kinetics of receptor expression (Figure 9A). In spite of this extensive analysis, we were not able to detect any noteworthy enhancement of EGF receptor mRNA levels. Hence, influences on EGFR trafficking seemed more likely. Immunocytochemistry revealed an enhanced EGFR signal at a SiO_2_ NP area concentration of 93.8 µg/cm^2^ (Figure 9B), which is in accordance with the results of the Western blot (Figure 7). Stimulation of the EGFR pathway induces receptor aggregation on the cell surface to enhance the chances of dimer formation and subsequently auto-phosphorylation [55,56]. The results of the present study suggest that the examined SiO_2_ NPs may induce a concentration-dependent receptor recruitment. Since no effect was detectable at the level of transcription, the effect of the NPs on the cellular growth might be due to a more “direct” effect mediated by the interaction of the silica particles with the cell membrane. The 12 nm SiO_2_ NPs are taken up by HT29 cells [28] and it is reasonable to assume that they are also internalized by GXF251L cells. In this respect, even though the exact uptake mechanism is still unclear, endocytosis seems to be most likely. In fact, SiO_2_ NPs were previously found to be internalized in a clathrin- or caveolin-dependent manner [17,57] or by passive cellular uptake due to an adhesive interaction of 14 nm SiO_2_ NPs with lipid membranes [27]. Activation of EGFR can presumably occur by different cellular mechanisms but its signaling capacity is strongly dependent on sorting and targeting for lysosomal degradation and its recycling to the plasma membrane [58,59]. Furthermore, substantial amounts of EGFRs and ligands can accumulate intracellularly due to the fact that the rate of receptor internalization is much faster than its degradation rate [60]. In this respect, it is possible to speculate that the uptake mechanisms of the NPs through cell membranes may lead to an impairment of the EGFR trafficking and homeostasis, causing, as a final effect, an alteration of the cellular proliferation and growth stimuli.

In spite of that, downstream responses seem to depend on particular cellular sites. That silica nanoparticles increase cell proliferation dependent on ERK1/2 activation is a valid hypothesis. However, the kinetics and dose-response in the various assays monitoring effects on growth and signaling varied. Complex cellular interactions are responsible for different signaling outcomes also in the case of ERK1/2 possibly resulting in both, anti- but also pro-apoptotic effects [61]. Here, further investigations are in progress to clarify this aspect in our cell system.

Altogether, even if it is difficult to predict the relevance of the SiO_2_ NP concentrations for daily dietary uptake due to different study results and estimations, we consider our results important toward a better comprehension of their biological effects on the GI level. The “Scientific Panel on Dietetic Products, Nutrition and Allergies” of the “European Food Safety Authority” estimated a typical intake of silicon of 20 to 50 mg/day in a 60 kg adult (0.3–0.8 mg/kg bodyweight/day) [43] which equals around 43 to 107 mg SiO_2_ per day (0.7–1.8 mg/kg bodyweight/day). Dekkers et al. [44] estimated a comparable intake value according to analyzes of silica additives in food products. Including these values, Yang et al. [25] deduced an daily exposure dose of the intestine of around 0.01–0.05 µg/cm^2^. The interior gastric surface area of adults with normal body weight is assumed to be 383 cm^2^ up to 800 cm^2^ in total [62,63]. Consequently, the gastric exposure dose would be around 54–279 µg/cm^2^. Further, an excess uptake of products containing (nano-) silica particles must be considered and additionally, the conjecture that probably not all food products which may contain E551 were identified in the respective study [44]. Therefore, Dekkers et al. [44] estimated an elevated uptake of 9.4 mg/kg bodyweight which would result in an enormous gastric exposure of 705–1473 µg/cm^2^ calculated for a 60 kg adult, distinctly exceeding the applied concentrations in the present study. Yet, current investigations suggest a daily intake of 5 mg/kg bodyweight as harmless throughout lifetime [64]. However, these amounts of intake are assumed to be safe [43,65] but it must be considered the given data refer to silicon and silica in general without taking into account nano-sized types of SiO_2_. Analyses of products containing E551 indicated a nano-silica content up to 43% [44]. Nonetheless risk assessment is difficult, thus enforcing, in our opinion the need for characterization of the nano-sized material.

All in all, the presence, behavior and bio-distribution, as well as the excretion of nano-sized materials have been extensively studied in the last decade. Thereby, oral uptake and with it the different gastrointestinal digestion stages received attention only in the recent years. Different test systems were applied to assess safety or hazard of silica-containing food, like in vitro [25,28] and in vivo [66,67,68,69] toxicity screenings or simulation of the gastrointestinal status and barrier [41,70]. However, it is not yet clear how amorphous SiO_2_ NPs influence health after oral uptake and if they can indeed per se be considered as safe in food products.

In conclusion, the examined food-grade silica particles with a nominal primary diameter of 12 nm are strongly agglomerated and aggregated in the applied suspension. They exert slight cytotoxic effects, as well as, growth and proliferation stimulation in gastric cells, which however, displayed differences in comparison to the effect on other cell lines [25,28]. Toxic effects were limited, but detectable in a time- and concentration-dependent manner. The stimulation of proliferation in the gastric carcinoma GXF251L cell line could not be attributed to the classical activation of the EGFR and/or ERK 1/2 signaling pathways, usually reflected by enhanced phosphorylation. However, depending on incubation time and applied concentration, both signalling elements were clearly influenced by the administered particles. Further, influences on receptor trafficking and homeostasis are assumed, whereby impact on transcription could be ruled out. However, particle characterization and the comparison with bigger particles indicate that the observed effects cannot necessarily be attributed to particles below 100 nm and thus might not be regarded as a “nano-specific” effect *per se*. However, it has to be pointed out, that the examined silica particles enhanced proliferation of the studied gastric carcinoma cell line, suggesting a potential risk for gastric cancer patients and underlining the necessity of further studies to clarify whether non-transformed gastric cells might be targeted as well.

## 4. Materials and Methods

### 4.1. Cell Culture

Experiments were performed using the established gastric cell line GXF251L (kindly provided by H. H. Fiebig, Institute for Experimental Oncology, Oncotest GmbH, Freiburg, Germany). GXF251L cells are a human tumor xenograft cell line derived from a signet ring cell carcinoma and were cultivated in Roswell Park Memorial Institute (RPMI) Medium 1640 containing l-glutamine. The media for GXF251L cells was supplemented with 25 mM HEPES (pH 7.2), 50 µg/mL gentamycin sulfate and 9% (*v*/*v*) heat-inactivated fetal bovine serum (FBS). To ensure homogeneous response and growth, cells were kept in culture for maximum 25 passages in humidified incubators with 5% CO_2_ at 37 °C. Media and supplements were purchased from Gibco^®^ Life Technologies™, Karlsruhe, Germany.

Cells were routinely tested for mycoplasm contamination and found to be negative. For all experiments only a cell density of around 70% to 80% confluence was selected for particle application. Exceptions were made for the microscopy-based determination of cell counts, apoptosis and necrosis and for the sulforhodamine B assay for which a confluence of around 40% to 50% was chosen to guarantee exponential cell growth over an extended incubation time of 24 h to 72 h.

### 4.2. Particles and Incubation Conditions

The commercially available, engineered amorphous silica nanoparticles Aerosil^®^ 200 F (hydrophilic, pyrogenic), especially designed as supplement for food and feed, were a kind gift from Evonik Industries (Essen, Germany). According to the manufacturer, the particle’s specific surface area is indicated as 200 ± 25 m^2^/g (BET method) and their nominal primary diameter as 12 nm. For the purpose of comparison, bigger SiO_2_ particles (Ångström-Sphere™) with a nominal primary diameter of 200 nm were included for some assays. According to the manufacturer Fibre Optic Centre Inc. (New Bedford, MA, USA), their specific surface area, also determined by the BET method, is given as 4 ± 2 m^2^/g and they are claimed to be applicable in food industry as well.

Previous studies conducted by Gehrke et al. [28] with the same 12 nm SiO_2_ particles revealed a broad particle size distribution and diameters of 16 to 40 nm of individual particles examined by transmission electron microscopy (TEM). TEM images and dynamic light scattering (DLS) analysis showed the presence of agglomerates and/or aggregates with a Sauter mean diameter (SMD) of more than 500 nm in FBS-containing Dulbecco’s Modified Eagle Media (DMEM). The suspension of the 200 nm SiO_2_ particles resulted in an instant formation of micrometer-sized agglomerates and aggregates which sedimented quickly. TEM analysis further revealed individual particles of sizes around 200 nm in the suspended fraction which was also confirmed by DLS measurements and was in accordance with the manufacturer’s information. Interestingly, neither the suspension medium (water or DMEM) nor the addition of low or high FBS-amounts seemed to influence the diameter of the 200 nm particles substantially. For both particles suspended in high-FBS containing DMEM, ζ-potentials of around—10 mV revealed quite unstable suspensions with a strong agglomeration/aggregation tendency. Further information regarding the agglomeration behavior as well as a detailed particle characterization in DMEM and in water have been published by Gehrke et al. [28]. In order to exclude specific influences of the particle behavior due to a differently applied cell culture medium, a detailed characterization of the 12 nm NPs suspended in FBS-containing RPMI 1640 medium was performed for the present study.

The particles were applied to cells according to a specific internal standard operating protocol (SOP) to guarantee equal treatment and incubation conditions between the different test systems. A stock suspension of 10 mg/mL was prepared by weighing 20 to 30 mg of the particles and addition of an appropriate volume of complete media, including a standard concentration of 9% FBS to avoid effects related to cytotoxicity or stress. All suspensions and dilutions were prepared in the respective cell culture media (RPMI 1640). The stock suspension was carefully vortexed and sonicated for 30 strokes with a Branson Sonifier^®^ S-450A (230 V ± 10 V, 50/60 Hz and 400 Watts with the following settings: duty cycle: 50%; output control: 4; including microtip 102C). Afterwards, the stock suspension was diluted 1:10 (*v*/*v*) gaining a particle suspension with a concentration of 1 mg/mL. Subsequently, the incubation suspensions in the range of 0.03 µg/cm^2^ up to 156.3 µg/cm^2^ (equivalent to 0.1 µg/mL up to 500 µg/mL) were prepared. Area and volume concentration conversions are based on a 96-well plate with a surface area of 0.32 cm^2^ and an application volume of 100 µL and scaled upon need (for more detailed information refer to Gehrke et al. [28]). Therefore, the applied volume varied depending on the area of the dish. Treatment of control cells was the same except for incubating the cells with the respective culture media without particles.

### 4.3. Nanoparticle Tracking Analysis

For analysis of particle size and particle size distribution (PSD) a state-of-the-art method, namely nanoparticle tracking analysis (NTA) was performed with the NanoSight NS500 (Malvern Instruments Ltd, Malvern, UK). Thereby, a laser beam was passed through a chamber containing the sample. The present particles moving under Brownian motion scattered the light and single particles could be tracked by means of a microscope equipped with a camera. Background and camera levels were set manually in dependency of the particle intensity and finally, the hydrodynamic diameters and PSDs were analyzed by the software (NTA 3.1, Malvern Instruments Ltd, Malvern, UK) using the Stokes-Einstein equation. In comparison to DLS, a bias of particle intensity is avoided and the resolution of particle sizes is improved [38].

Nevertheless, preliminary experiments revealed a limitation of NTA. The measurement of the incubation suspensions as described in Section 2.1 resulted in high background levels, strong particle flares outshining smaller particles and subsequently, in vague, deviating values—an issue owed to the high amounts of FBS (9%). In consequence, the FBS-amount was adjusted to the highest necessary volume, which was 2.7% for the analyzed suspension with an area concentration of 93.8 µg/cm^2^. The 12 nm SiO_2_ NPs were pre-suspended in 9%-FBS containing RPMI 1640 medium (including 1% HEPES) and also double-distilled water with a concentration of 10 mg/mL as described beforehand. The suspensions were diluted to 1 mg/mL with 9%-FBS containing medium or double-distilled water, respectively and further diluted with medium to the final incubation concentrations of 31.3 and 93.8 µg/cm^2^. For the latter dilution, FBS was added separately, resulting in various final amounts of 0.9% and 2.7% in order to discern possible FBS-related effects. The suspensions were analyzed shortly after preparation (0 h) and after 24 h of incubation at 37 °C. For NTA measurements, the suspensions were diluted with double-distilled water in a manner that single particles could be discerned and were analyzed after maintaining the temperature of the sample chamber to 25 °C with individually set background and camera levels. In order to avoid drift of the samples and to ensure reproducible results, the chamber was pre-loaded with the dilution medium (RPMI 1640 in water) prior to and in-between each analysis until not more than 5 particles were recorded. Five different frames, 60 s each, were recorded and merged by the software. The mean diameter ± SD of at least two independent experiments was calculated and the PSD was considered individually.

### 4.4. ζ-Potential

The dispersion of particles may probably result in the formation of agglomerates and/or aggregates. The re-agglomeration behavior and thus, the stability of the particle suspensions was analyzed also by means of ζ-potential measurements (Malvern Zetasizer Nano-ZS, Malvern Instruments Ltd, Malvern, UK). The method is based on laser Doppler electrophoresis and particle movement under Brownian motion due to an applied electric field. Thereby, the moving particles scatter the light of a laser beam passing through the suspension resulting in fluctuations of the light intensity. Since the frequency shift is proportional to particle velocity, particle motion can be calculated by the software (Zetasizer software v7.11, Malvern Instruments Ltd, Malvern, UK) from the ratio of velocity to electric field strength. The 12 nm SiO_2_ NP stock suspensions (1 mg/mL) were prepared in double-distilled water and RPMI 1640 medium (containing 1% HEPES and 9% FBS). The incubation suspensions with area concentrations of 31.3 and 93.8 µg/cm^2^ were pre-suspended in medium and examined with regard to different final FBS amounts (see also Section 2.1). The samples were analyzed without further dilution inside of a disposable capillary cell with electrodes shortly after sample preparation (0 h) and after 24 h of incubation at 37 °C. The ζ-potential was recorded in triplicates after sample equilibration to 25 °C. The mean of the triplicates and subsequently, the mean ± SD of at least two independent experiments was calculated.

### 4.5. Microscopy-Based Determination of Cell Counts, Apoptosis and Necrosis

In order to obtain a direct evaluation of the cytotoxic potential of the 12 nm, as well as, 200 nm SiO_2_ particles, a high-content-high-throughput screening assay covering multiple endpoints at the single cell level was chosen. The microscopic determination of total and viable cell counts, as well as, the distinction of different types of cell death was performed according to the method published in Donauer et al. [71] with slight modifications. The number of seeded cells for each treatment was chosen depending on the final particle incubation time as described above in Section 2.2 cell culture to ensure homogeneous and comparable cell density during the treatment. For this experimental set-up, incubation times of 4 h, 24 h and 72 h were chosen. Cells were allowed to adhere for 23 h achieving a confluence of approximately 70% (4 h of incubation) and 50% (24 h and 72 h of incubation), respectively, at the beginning of the incubation period. The pure incubation media served as a negative control and staurosporine (800 nM) was included as a positive control. Thereafter, cells were stained with 0.3 µg/mL Hoechst33342 and 0.25 µg/mL propidium iodide (PI) and images were acquired with the help of the Olympus IX-81-ZDC microscope and the ScanR Acquisition software. Finally, the data were analyzed with the ScanR Analysis software (OLYMPUS, Hamburg, Germany).

Every concentration was applied as quadruplicate at least and every experiment was performed three times using cells from different passages and/or cell culturing flasks. Per well four images were taken for evaluation resulting in a total of 48 replicates per treatment. The effects on cellular growth were determined as percentage of total cell count (treated-over-control ratio × 100%) and percentage of viable, apoptotic and necrotic cells in relation to total cell count (treated-over-control ratio × 100%), respectively.

### 4.6. Cytotoxicity

The influence of the 12 nm SiO_2_ NPs on cell proliferation and cytotoxicity was determined by four different bulk assays measuring various endpoints like mitochondrial activity, membrane integrity and protein content/cell number. This approach was chosen in order to verify the data and to reduce the risk of experimental artifacts.

#### 4.6.1. Water Soluble Tetrazolium (WST-1) Assay

In order to determine the effects on cell toxicity and viability, the commercially available WST-1 test kit was used (Roche Applied Science, Mannheim, Germany). The test principle is based on the ability of cells to reduce the tetrazolium salt 4-[3-(4-iodophenyl)-2-(4-nitrophenyl)-2*H*-5-tetrazolio]-1,3-benzene disulfonate to a water soluble formazan salt, which can be measured in a plate reader at λ = 450 nm. Cells were seeded into a 96-well plate and allowed to grow for 48 h (cell confluence of around 70%). Thereafter, cells were incubated with the respective SiO_2_ NPs prepared according to the SOP for 45 min or 24 h. Triton X-100 was included in the test system as a positive control. Then, cells were washed twice with 100 µL PBS and incubated with 110 µL of a 1:10 WST-1 reagent/medium dilution (*v*/*v*). Subsequently, the plates were measured including a reference wavelength of λ = 650 nm. Each SiO_2_ NPs preparation was tested three times with cells from at least three different passages and every concentration was measured six times resulting in eighteen replicates for each experimental point. The effect on the mitochondrial activity was expressed as percentage of viability (treated-over-control ratio × 100%).

#### 4.6.2. Lactate Dehydrogenase (LDH) Assay

In parallel to the WST-1 assay, the potential for cytotoxicity of SiO_2_ NPs was also determined by measuring the lactate dehydrogenase release in the cell medium. To this aim the commercial LDH kit (Roche Applied Science, Mannheim, Germany) was used. In the case of cell lysis, released LDH catalyzes in a coupled enzymatic reaction the reduction of the tetrazolium salt 2-[4-iodophenyl]-3-[4-nitrophenyl]-5-phenyltetrazolium chloride (INT) to a red colored, water soluble formazan salt, which enables the correlation of the spectrophotometric signal with the cell status. After particle incubation, 50 µL of the supernatant and 50 µL of PBS were carefully transferred into a clean 96-well plate for the LDH assay. Then 100 µL of the freshly prepared reaction mixture (according to manufacturer’s handbook) were added to every well. The plates were incubated under agitation for 5 min at room temperature and measured immediately with a plate reader at λ = 490 nm. A reference wavelength (λ = 650 nm) was recorded and subtracted. For each particle preparation, tests were carried out at least three times using cells from at least three different cell preparations. Each silica concentration was measured six times resulting in eighteen replicates. The effects on the LDH release were determined as percentage of LDH leakage (treated-over-assay control ratio × 100%).

#### 4.6.3. Sulforhodamine B (SRB) Assay

To determine the influence on cellular growth, the SRB assay was performed according to Skehan et al. [72] with slight modifications. Therefore, cells were seeded into 96-well plates and allowed to grow for 48 h prior to treatment up to 40% of confluence. Cells were incubated with SiO_2_ particle suspensions for 24 h, 48 h or 72 h, respectively, in complete medium. Incubation was stopped by addition of 10 µL of trichloroacetic acid (50% *v*/*v* solution). After 1 h at 4 °C, plates were washed four times with water. The dried plates were stained with a 0.4% (*w*/*v*) solution of SRB. Again, plates were washed twice with water and acetic acid solution (1% *v*/*v*). The dried protein-bound dye was dissolved with 100 µL Tris-buffer (10 mM, pH 10.5) and quantified photometrically (λ = 570 nm). The effects on cellular growth were determined as percentage of survival (treated-over-control ratio × 100%).

### 4.7. Western Blot Analysis

Phosphorylation of the ERK 1/2 as well as the phosphorylation of the EGFR were analyzed by Western blot according to the method prior published by Gehrke et al. [28]. In brief, GXF251L cells were seeded into Petri dishes (Ø 10 cm) using 10 mL of the respective growth media supplemented with 9% FBS and allowed to grow for 96 h. Cells were starved in low-serum medium for additional 24 h to arrest cell growth and to equalize cell cycle phase. Thereafter, cells were treated with the 12 nm SiO_2_ NP suspensions for 45 min or 24 h. After the incubation period, cells were stored on ice and cell lysis was performed using a commercially available Cell Lysis Buffer (10× stock; New England BioLabs GmbH, Frankfurt, Germany) including 1 mM PMSF. Protein content was adjusted by means of Bradford assay and subsequently, proteins were separated by SDS-PAGE and transferred onto a nitrocellulose membrane by Western blot. After blocking in 5% (*w*/*v*) milk powder diluted with Tris-buffered saline containing 0.1% Tween-20, membranes were incubated using rabbit antibodies against human EGFR (polyclonal, detection of total EGFR protein), pEGFR (polyclonal, detection of EGFR only when phosphorylated at Tyr1068), p44/42 MAPK (ERK1/2) (monoclonal, detection of total p44/42 MAP kinase protein) and phospho-p44/42 MAPK (pERK1/2) (polyclonal, detection of p44/42 MAP kinase when individually or dually phosphorylated at Thr202 and Tyr204 of ERK1/Thr185 and Tyr187 of ERK2), all purchased from Cell Signaling Technology (provided by New England BioLabs GmbH, Frankfurt, Germany). α-tubulin (mouse monoclonal, raised against amino acids 149–448 of α-tubulin of human origin; Santa Cruz Biotechnology, Inc., Heidelberg, Germany) was included as a loading control and detected on the same membrane after cutting it in two pieces. Anti-rabbit as well as anti-mouse IgG horseradish peroxidase conjugates (Santa Cruz Biotechnology, Inc., Heidelberg, Germany) were used as secondary antibodies. The respective chemoluminescent signals (LumiGlo Reagent & Peroxide: Cell Signaling Technology provided by New England BioLabs GmbH, Frankfurt, Germany) were analyzed using the LAS 4000 system with the Multi Gauge Image Analyzer software version 3.2 for quantification (Fujifilm, Tokyo, Japan). Arbitrary light units were plotted as treated-over-control ratios (T/C × 100%).

### 4.8. Immunocytochemistry

Immunocytochemical (IC) experiments were performed for the localization of Ki-67 and the EGF receptor. In the first case, GXF251L cells were incubated with the respective concentrations of 12 nm SiO_2_ NPs for 24 h and in the latter for 45 min following the same protocol of seeding and incubation already described for the Western blot experiments. At the end of the incubation time cells were washed with PBS and fixed with 3.7% formaldehyde (15 min, room temperature). After fixation, cells were washed, permeabilized with 0.2% Triton X-100 and blocked with 1% BSA (Ki-67) or 10% normal goat serum (NGS; EGFR) (1 h, room temperature). For the IC analysis, Lamin B goat polyclonal antibody (sc-6216), Ki-67 rabbit polyclonal antibody (sc-15402) and α-tubulin mouse monoclonal antibody (sc-5286) (all purchased from Santa Cruz Biotechnology, Inc., Heidelberg, Germany) were used. In addition, a rabbit polyclonal antibody against human EGFR was used (New England BioLabs GmbH, Frankfurt, Germany). Fluorescent secondary antibodies raised against relevant species were then used for the detection according to manufacturer details (Alexa Fluor 647 Anti-Goat, Alexa Fluor 568 Anti-Rabbit and Alexa Fluor 488 Anti-Mouse antibodies, Life Technologies, Vienna, Austria). After incubation with the secondary antibodies cells were washed and post-fixed with 3.7% of formaldehyde and free aldehyde groups were then quenched with PBS glycine (100 mM). Slides were mounted and sealed with Roti-Mount FluorCare (Roth, Graz, Austria) and imaged after 48 h with a Confocal LSM Zeiss 710 meta equipped with a Plan Apochromat 63X/1.4 oil objective, zoom 2. For image analysis and quantification, the software Zeiss ZEN was used. Images were acquired from three different cell preparations keeping the acquisition parameters constant within the experimental session (Laser power, gain, background correction). Image quantification was performed in the central section of the z-stack of the nucleus and for every experimental condition more than 60 nuclei were quantified and signal intensities were evaluated as treated-over-control ratios (T/C × 100%).

### 4.9. RNA Extraction and qRT-PCR

For RNA extraction, GXF251L cells were seeded into Petri dishes (Ø 6 cm) and allowed to grow to a confluence of around 70%–80%. Thereafter, cells were treated with the respective 12 nm SiO_2_ NPs suspensions for 2 h, 6 h, 16 h and 24 h, prepared according to the SOP and harvested subsequently. Total RNA was extracted with the QuantiTect RNeasy Mini Kit (Qiagen GmbH, Hilden, Germany) and RNA content was determined with the Nanodrop 2000c (Peqlab). The QuantiTect Reverse Transcription Kit (Qiagen GmbH, Hilden, Germany) along with the Peltier Thermal Cycler (BioRad) were used for reverse transcription of the extracted RNA into cDNA. Subsequently, transcripts were amplified and quantified by the StepOne Plus SYBR-Green based quantitative real-time PCR (Life Technologies, Applied Biosystems, Vienna, Austria) by means of the QuantiTect SYBR Green PCR Kit and QuantiTect Primer Assay (Qiagen GmbH, Hilden, Germany). Thereby, the EGFR (Hs_EGFR_1_SG) gene was analyzed and β-actin (Hs_ACTB_1_SG), as well as, GAPDH (Hs_GAPDH_2_SG) were analyzed as housekeeping genes. No-template controls and melting curves were included to exclude potential contaminations and non-specific amplifications. The respective relative transcription rates were normalized to the housekeeping genes and then calculated using the ΔΔCT-method.

### 4.10. Statistics

Unless stated otherwise, all results are given as mean value of at least three different independent experiments ± standard deviation (SD) and are depicted as treated-over-control T/C [%]. Data were analyzed using the OriginPro 9.1.0G software (OriginLab Corporation, Northampton, MA, USA). Tests for normal distribution were performed by Shapiro-Wilk test (*p* ≥ 0.05). For statistical analysis of differences of the mean between the several tested groups One-way ANOVA and subsequently “Fisher’s Least Significant Differences (LSD)” post-hoc test were performed. “Student’s *t*-test for two independent samples” was performed to compare significances between only two groups. Symbols are used in figures to indicate a comparison to the respective negative control with statistical levels of *p* ≤ 0.05 for *, *p* ≤ 0.01 for ** and *p* ≤ 0.001 for ***. Bold printed letters in a figure indicate a significant difference of these data and were considered statistically significant with a significance level of at least *p* ≤ 0.05.

## Figures and Tables

**Table 1:** Mean diameters ± standard deviations (SD) measured by nanoparticle tracking analysis and ζ-potentials of various 12 nm SiO_2_ particle suspension preparations comprising different amounts of fetal bovine serum (FBS) analyzed shortly after sample preparation (0 h) and after 24 h of incubation.

12 nm SiO_2_ NP Suspensions			Mean Diameter ± SD [nm]		ζ-Potential [mV]	
Designation	Particle concentration	Suspension media (final FBS-content)	0 h	24 h	0 h	24 h
stock suspension	1 mg/mL	RPMI 1640 (9% FBS)	336 ± 7	301 ± 58	−11.5 ± 0.3	−11.3 ± 0.4
	1 mg/mL	dd water (FBS-free)	224 ± 17	220 ± 27	−33.1 ± 6.6	−25.0 ± 5.0
incubation suspension	31.3 µg/cm^2^ (100 µg/mL)	RPMI 1640 (0.9% FBS)	333 ± 26	317 ± 20	−11.0 ± 0.0	−12.0 ± 0.4
		RPMI 1640 (2.7% FBS)	323 ± 29	261 ± 98	−9.9 ± 0.9	−10.3 ± 0.0
		RPMI 1640 (9% FBS)	-	-	−9.5 ± 0.3	−9.3 ± 0.6
	93.8 µg/cm^2^ (300 µg/mL)	RPMI 1640 (2.7% FBS)	315 ± 1	314 ± 25	−11.8 ± 0.2	−11.3 ± 0.1
		RPMI 1640 (9% FBS)	-	-	−10.5 ± 0.1	−10.0 ± 0.5

## Abbreviations

The following abbreviations are used in this manuscript:

JNK1/2	c-Jun NH_2_-terminal kinases1/2
DMEM	Dulbecco’s Modified Eagle Medium
DSMZ	German Collection of Microorganisms and Cell Cultures
EGFR	epidermal growth factor receptor
ERK1/2	extracellular signal-regulated kinases 1/2
FBS	fetal bovine serum
Fisher’s LSD	Fisher’s Least Significant Differences
NTA	nanoparticle tracking analysis
GIT	gastrointestinal tract
LDH	lactate dehydrogenase
MAPK	mitogen-activated protein kinases
PI	propidium iodide
PSD	particle size distribution
qRT-PCR	quantitative real-time polymerase chain reaction
RPMI 1640	Roswell Park Memorial Institute Medium 1640
SiO_2_ NPs	silica nanoparticles
SRB	sulforhodamine B
SOP	standard operating procedure
T/C	test over control
WST-1	water soluble tetrazolium
